# Functional Characterization of *Eccyp307a1* in Early Ovary Development of *Exopalaemon carinicauda*

**DOI:** 10.3390/ijms27031481

**Published:** 2026-02-02

**Authors:** Shaoting Jia, Xiaotong Pan, Yashi Hou, Kezhi Gong, Yichen Su, Jianjian Lv, Jitao Li

**Affiliations:** 1 State Key Laboratory of Mariculture Biobreeding and Sustainable Goods, Yellow Sea Fisheries Research Institute, Chinese Academy of Fishery Sciences, Qingdao 266071, China; 2 Laboratory for Marine Fisheries Science and Food Production Processes, Laoshan Laboratory, Qingdao 266237, China; 3 National Experimental Teaching Demonstration Center for Aquatic Sciences, Shanghai Ocean University, Shanghai 201306, China

**Keywords:** *Exopalaemon carinicauda*, cytochrome P450 family, CYP307A1, crustaceans

## Abstract

According to a previous study, in insects, *cyp307A1* plays a central role in ecdysteroid synthesis, which is a member of the cytochrome P450 family. However, the function of *cyp307A1* in crustaceans remains unclear. In this study, we explored the function of *Eccyp307a1* in *Exopalaemon carinicauda* through a series of experiments. The sequence of *Eccyp307a1* encoded 529 amino acids, and the protein was found to possess typical P450 domains and heme-binding sites. The mRNA of *Eccyp307a1* was expressed at a higher level during the early stages of ovary development, but was expressed less during the mature stage. Furthermore, employing eyestalk ablation and RNAi experiments, we determined that *Eccyp307a1* could be regulated by neuroendocrine factors and is essential for the normal initiation of ovary development. These findings provided insights into the gene function of *Eccyp307a1* in early ovary development in *E. carinicauda,* and our study further elucidates the molecular mechanisms of ovary development in crustaceans.

## 1. Introduction

The cytochrome P450 superfamily is of vital importance, with functions involved in redox reactions, environmental toxin metabolism, and hormonal regulation [1,2]. In insects, members of the CYP450 family are key components of the ecdysone biosynthesis pathway, which is crucial for regulating larval growth, development, and metamorphosis [3,4].

There are usually several key genes related to the ecdysone synthesis and regulation pathway, including CYP307A1/A2/B1, CYP306A1, CYP302A1, CYP315A1, CYP314A1, and CYP18A1 [5,6,7]. The first five kind genes are known as Halloween genes, initially discovered in Drosophila, and they primarily participate in ecdysone synthesis [8,9,10], while CYP18A1 is mainly involved in the regulation of ecdysone [11]. Among these genes, CYP307A1, which is known as spook, has been a focus in insect physiology and endocrinology research since its identification. Namiki et al. found that CYP307A1 played a decisive role in the early rate-limiting step of ecdysone biosynthesis, being primarily involved in the conversion of 7-dehydrocholesterol to Δ4-diketol. CYP307A1 was found to be specifically expressed in the prothoracic gland, which is essential for normal larval molting and metamorphosis [12]. Researchers further confirmed that silencing CYP307A1 leads to the severe disruption of ecdysone synthesis, inhibition of related signaling pathways, and a decrease in insect larvae survival rate [13].

With the development of the research method, additional roles played by ecdysone synthesis-related genes have gradually been revealed, extending beyond their classical roles in ecdysone synthesis. Scientists have discovered that the expression of CYP307A1 is not restricted to the prothoracic gland, which is the hormone-synthesizing organ; instead, it is also actively transcribed in other tissues, such as wing buds and the epidermis, during the embryonic stages [14]. Furthermore, the expression patterns of CYP307A1 exhibit significant variations across different species. Toshiki et al. found that cyp307a1 is expressed in insect ovaries [12]. More intriguingly, Wu et al. discovered that when the CYP307A1 function was inhibited, the developmental defect phenotype could only be partially rescued by exogenous supplementation of ecdysone, which suggested that cyp307a1 may have functions independent of ecdysone synthesis [14]. Several additional studies have supported this view. For instance, CYP307A1 was recently found to be involved in the diapause process of the silkworm, and its expression could be precisely regulated by the RNA epigenetic reader protein YTHDF3 [15]. Ardavan et al. found that CYP307A1 may be related to the cheliped regeneration of *Cherax quadricarinatus* [16]; Gong et al. discovered that the upregulation of CYP307A1 expression is associated with resistance to growth regulator insecticides in the tobacco cutworm [17]. The evidence provided by these studies collectively indicates that CYP307A1 might play multiple roles in broader developmental and adaptive regulatory networks. Similarly, other Halloween genes, such as CYP315A1, have also been found to have functions beyond ecdysone synthesis; for example, Jiang et al. discovered that CYP315A1 could promote ovarian development in *Macrobrachium nipponense* [18].

The *Exopalaemon carinicauda* is an important cultured shrimp species in China, primarily distributed in the southeast coastal areas. It has many advantages, including strong adaptability, a transparent body, and a short reproductive cycle [19], which are suitable for use in research. Ovary development in crustaceans is a core process in reproduction, involving complex hormonal regulation and gene expression networks. In our previous study, we found that *Eccyp307a1* was significantly highly expressed during the early stage of ovary development by dual color fluorescence in situ hybridization [20], suggesting that it might play a role in ovary development. To further elucidate the function of *Eccyp307a1* in *E. carinicauda*, we assessed the conservation of the *Eccyp307a1* amino acid sequence through phylogenetic analysis. Then the expression patterns, including different embryo developmental stages, various tissues, and different ovary development phases, were examined via RT-PCR and real-time quantitative PCR. Furthermore, the function of *Eccyp307a1* in the ovary was investigated through eyestalk ablation and RNA interference. The findings of this study expand our understanding of the functional diversity of *Eccyp307a1* and provide potential molecular targets for reproductive regulation and genetic breeding in crustaceans.

## 2. Results

### 2.1. Gene Structure of Eccyp307a1

**Figure 1:**
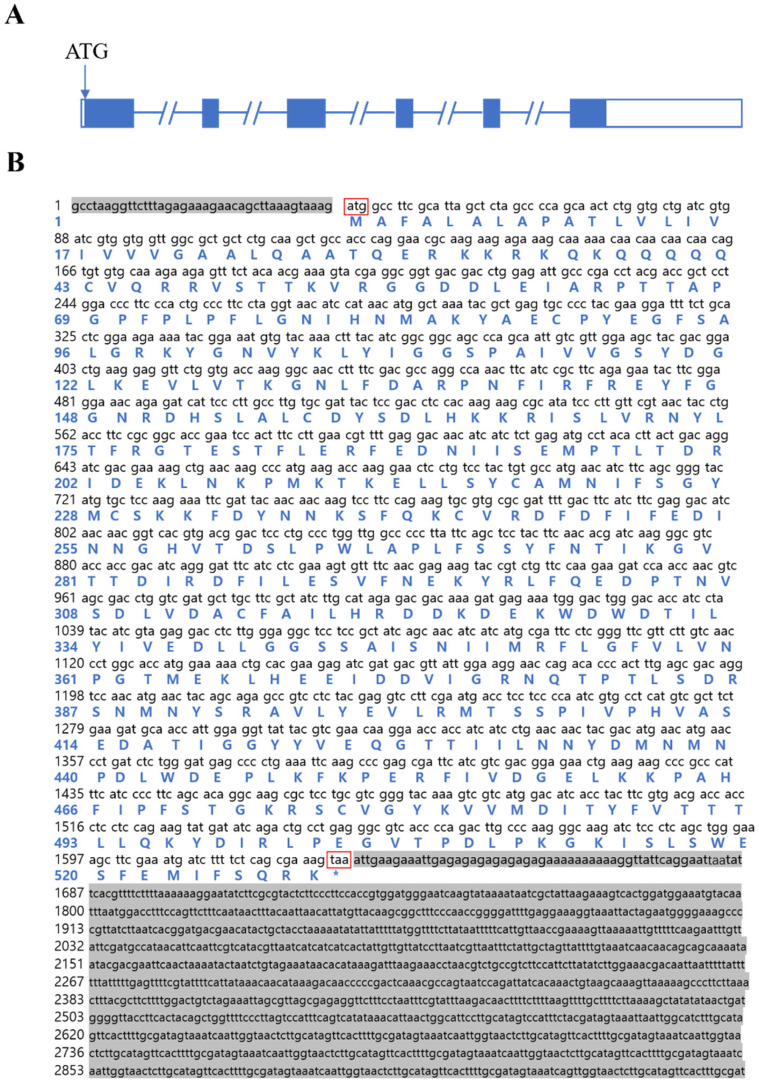
The gene structure and amino acid sequence of *Eccyp307a1*. (**A**) The gene structure of *Eccyp307a1*. (**B**) The amino acid sequence of *Eccyp307a1*. The gray regions mean 5′ UTR and 3′ UTR, respectively. Uppercase letters: amino acids, lowercase letters: cDNA sequences, numbers indicate sequence positions, and red boxes: stop codons. * stands for the termination site of amino acid translation.

The gene structure of *Eccyp307a1* was shown in Figure 1A, and it comprised 6 exons and 5 introns. The full-length cDNA of *Eccyp307a1* was 6734 bp, including a 5′ UTR of 39 bp, a 3′ UTR of 1342 bp, and a CDS of 1590 bp. The coding sequence began with the start codon ATG and ended with the termination codon TAA, which encoded a protein of 529 amino acids (Figure 1B).

### 2.2. The Conservation Analysis of Eccyp307a1

**Figure 2:**
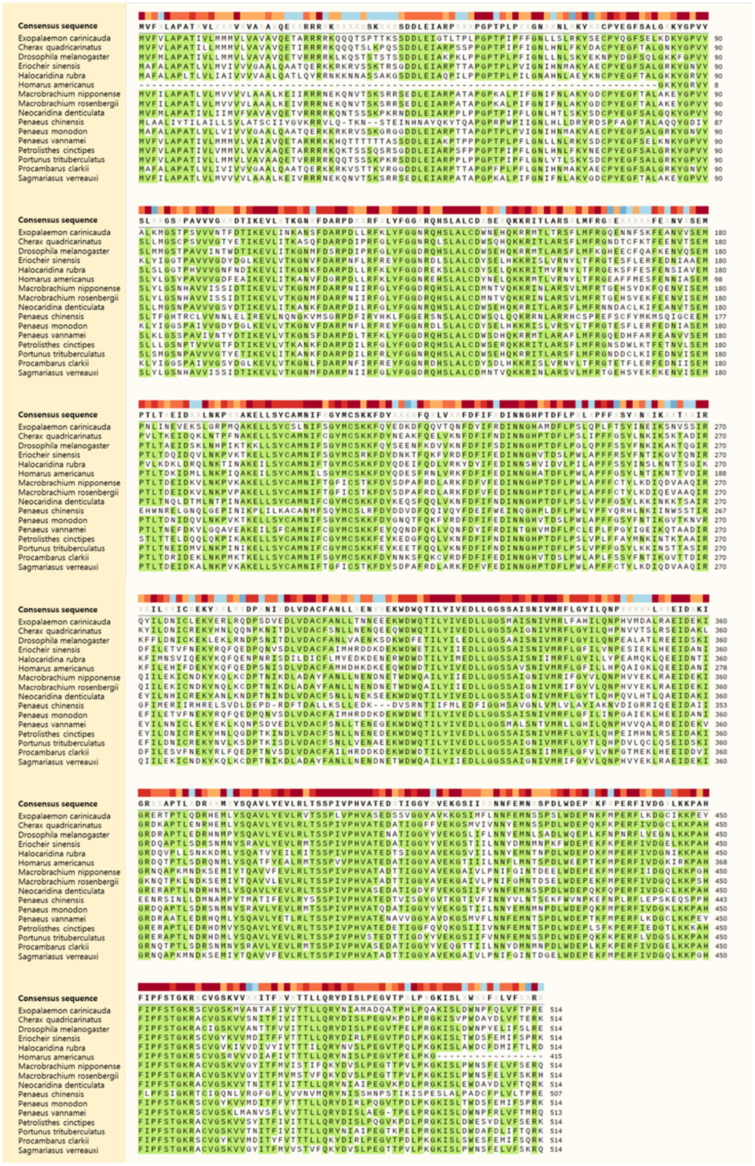
Protein sequence alignment of multiple species. Green indicates conserved amino acid regions, while white indicates non-conserved regions. And the numbers indicate sequence positions.

The results of the protein sequence alignment between *E. carinicauda* and other species are presented in Figure 2. This analysis delineated the conserved and variable regions within the homologous protein sequences. The N-terminal and C-terminal regions exhibit greater variability, suggesting they might be under fewer evolutionary constraints.

### 2.3. Phylogenetic Analysis

**Figure 3:**
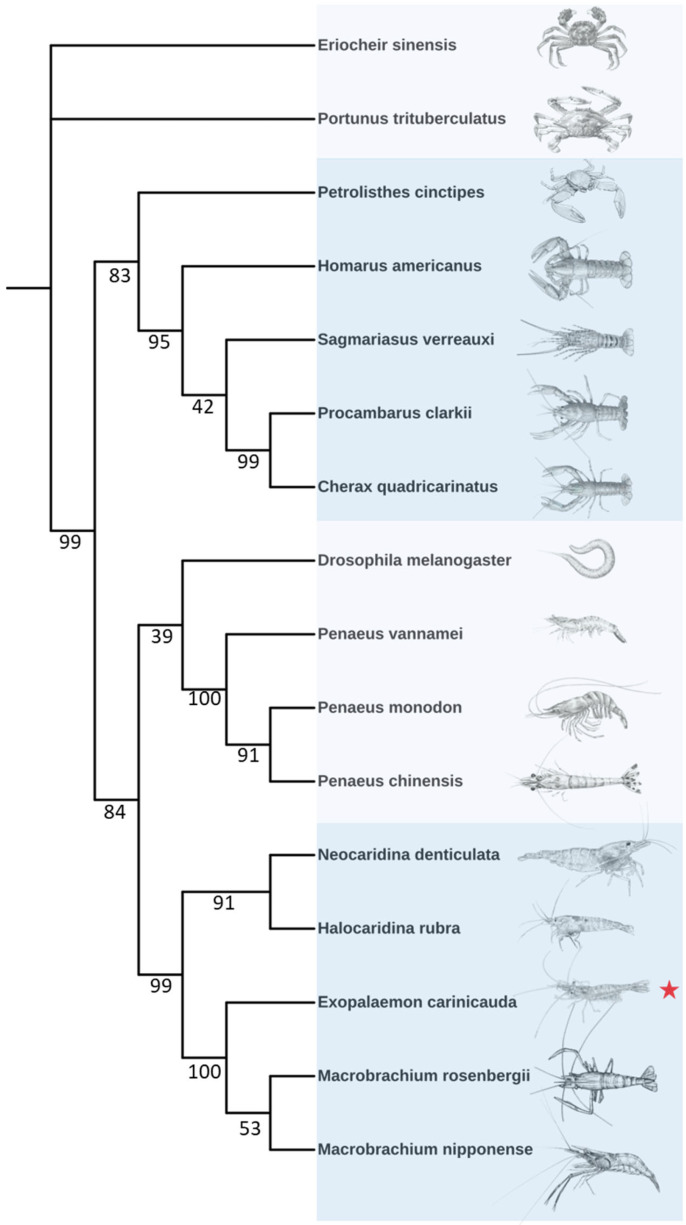
Phylogenetic tree of different species. The red star means the species in this study. The numbers means the bootstrap value and the same color block represents the same branch.

To clarify the phylogenetic relationship between *E. carinicauda* and other species, a phylogenetic tree was constructed based on the amino acid sequences of *Eccyp307a1*, as shown in Figure 3. The tree was primarily divided into two major clades. The upper clade predominantly consisted of crabs and crayfish, while the lower clade predominantly consisted of penaeid shrimps and prawns, indicating significant genetic divergence. *E. carinicauda* has the closest relationship to *M. nipponense* and *M. rosenbergii*, and the most distant relationship to *Drosophila melanogaster*.

### 2.4. Analysis of Functional Domains and Protein Structure

**Figure 4:**
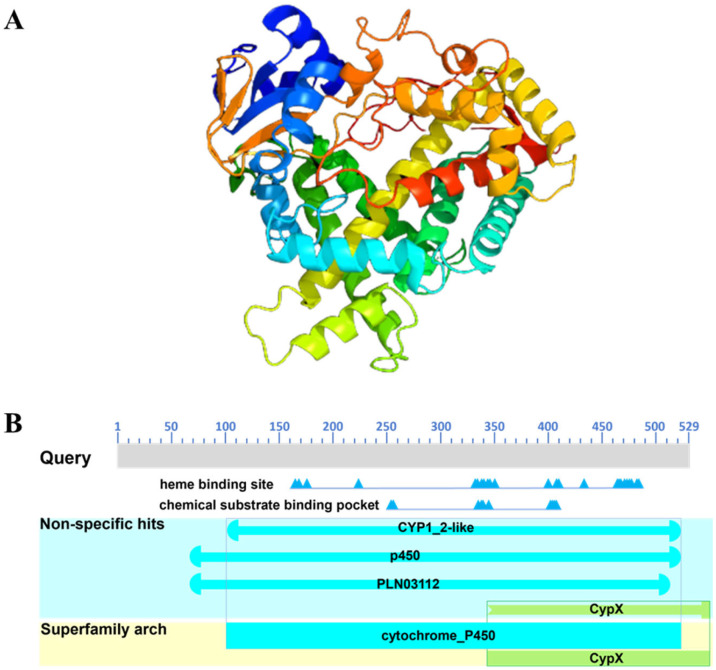
The 3D structure and domain distribution diagram of *Eccyp307a1*. (**A**) The 3D structure of *Eccyp307a1* protein. (**B**) The domain distribution diagram of *Eccyp307a1*. The light blue square indicates the domain of the protein.

The three-dimensional structural model of the *Eccyp307a1* protein is shown in Figure 4A, which contains two α-helices and two β-sheets. The predicted functional domains of *Eccyp307a1* were shown in Figure 4B. It possesses a typical cytochrome P450 domain, featuring a heme-binding site and a chemical substrate-binding pocket.

### 2.5. Spatiotemporal Expression Pattern of Eccyp307a1

**Figure 5:**
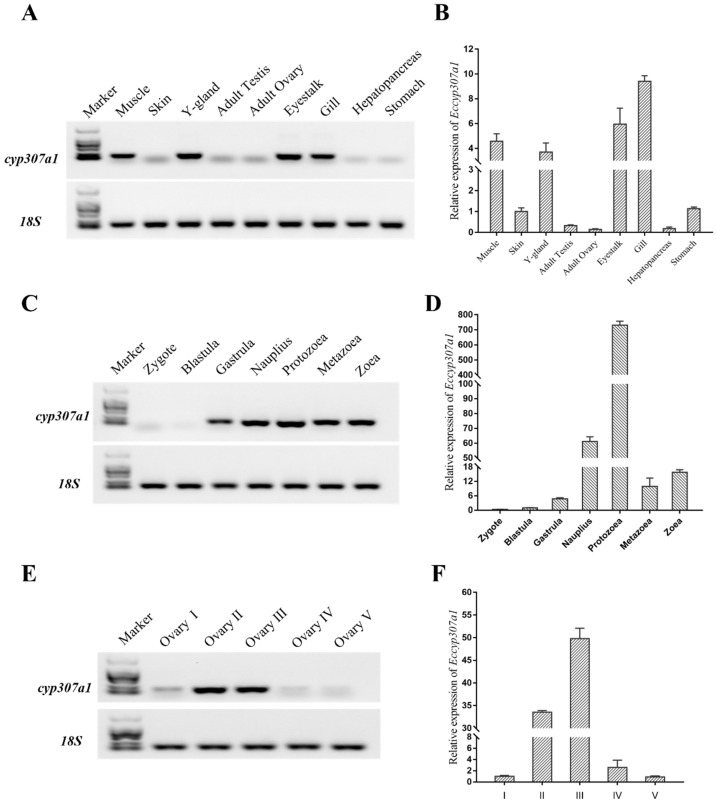
The expression pattern of *Eccyp307a1*. (**A**,**B**): The expression of *Eccyp307a1* in different tissues by RT-PCR and qPCR; (**C**,**D**): The expression of *Eccyp307a1* during the early embryo-developing stage by RT-PCR and qPCR; (**E**,**F**): The expression of *Eccyp307a1* in different stages of ovary development by RT-PCR and qPCR. The data was analyzed by the method 2^−ΔCT^, and the reference gene was *18S*.

To elucidate the expression pattern of the *Eccyp307a1* gene, RT-PCR and qPCR were performed (Figure 5). The results indicated that the *Eccyp307a1* expression levels were relatively high in muscle, Y-organ, eyestalk, and gill, and lower in other tissues. The expression of *Eccyp307a1* started from the gastrula stage and continued throughout the nauplius, protozoea, metazoea, and zoea stages. Regarding ovary development, *Eccyp307a1* expression was significantly elevated from stage II, but it was almost undetectable in nearly mature ovaries (stages IV and V).

### 2.6. The Expression Level of Eccyp307a1 After Eyestalk Ablation

**Figure 6:**
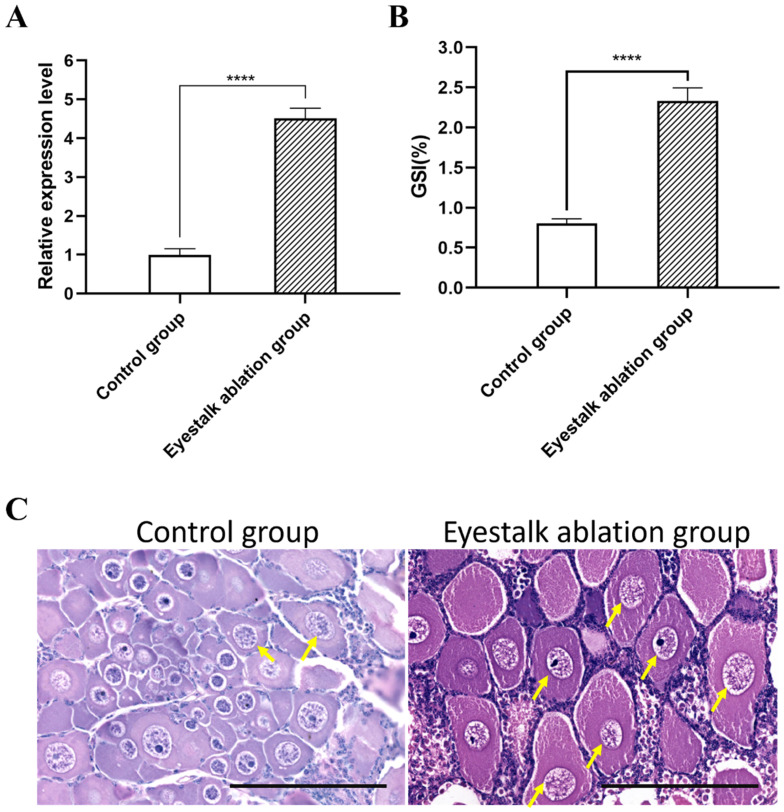
The expression pattern and ovary development after eyestalk ablation. (**A**) The expression level of *Eccyp307a1* by *real-time* PCR. The data was analyzed by the method 2^−ΔΔCT^, and the reference gene was 18S. (**B**) The gonadosomatic index (GSI) of different groups. (**C**) The paraffin section of the ovary, with a scale bar of 300 μm. The yellow arrows indicated the oocytes entered into pachytene stage in meiosis. ****, *p* < 0.0001.

To further investigate the function of the *Eccyp307a1* gene, unilateral eyestalk ablation was performed on *E. carinicauda*. The expression of *Eccyp307a1* was upregulated in the ablation group after 7 days. The gonadosomatic index (GSI) also significantly increased in the ablation group. There were a greater number of primary oocytes in the ablation group than in the control group (Figure 6). These results suggest that the *Eccyp307a1* gene might play a role in ovary development.

### 2.7. Functional Study of Eccyp307a1 via RNAi

**Figure 7:**
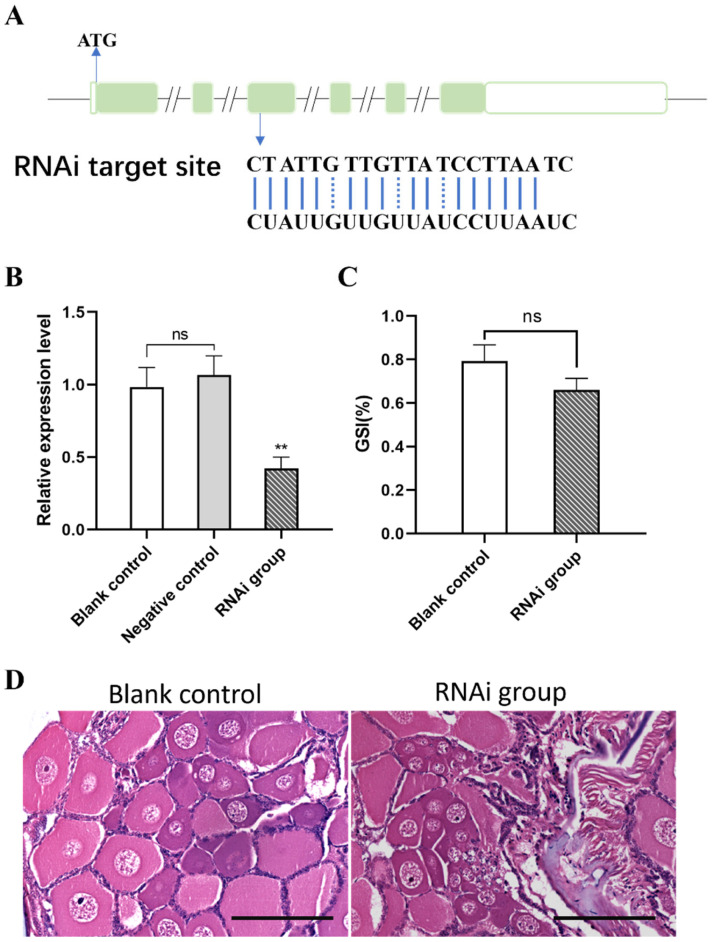
Knockdown of *Eccyp307a1* by RNAi. (**A**) The RNAi target site of *Eccyp307a1*. (**B**) The expression level of *Eccyp307a1* after RNAi by *real-time* PCR. The data was analyzed by the method 2^−ΔΔCT^, and the reference gene was 18S. (**C**) The gonadosomatic index (GSI) of different groups. (**D**) The paraffin section of the ovary, with a scale bar of 300 μm. **, *p* < 0.01; ns, no significance.

Subsequently, RNAi was employed to knock down *Eccyp307a1*, with the target siRNA designed against the third exon (Figure 7A). The expression of *Eccyp307a1* showed an obvious downregulation after RNA interference(Figure 7B), which indicated the knockdown was effective. However, statistical analysis revealed no significant difference in the GSI between the knockdown group and the control group (Figure 7C). We were able to identify a slight inhibition of ovary development via paraffin section in the RNAi group compared to the control (Figure 7D).

## 3. Discussion

In this study, we elucidated the spatiotemporal expression pattern of *Eccyp307a1* in *E. carinicauda* using RT-PCR and qPCR, revealing its high expression during early ovary developmental stages. The function of *Eccyp307a1* was investigated by eyestalk ablation and RNAi experiments. The results showed that *Eccyp307a1* could be regulated by endocrine organs such as the eyestalk, and its evolution and function exhibit both conservation and species-specific characteristics. Our findings could provide insights into understanding reproductive development in crustaceans.

### 3.1. Eccyp307a1 Expresses an Early Ovary Development Switch Regulated by Neuroendocrine Factors

In vertebrates, gene function is often closely associated with its expression location. Genes related to gonadal development are typically expressed in the gonads [21]. For example, in medaka, the *dmy* gene, a male sex determination gene, is specifically expressed in the testis [22]. In crustaceans, however, possibly due to their open circulatory system [23], many functionally specific genes exhibit a ubiquitous expression pattern across various tissues. For instance, in *Fenneropenaeus merguiensis*, the *IAG*, which is considered crucial for male sex differentiation, is expressed in multiple tissues [24]. Therefore, it is understandable that *Eccyp307a1* is expressed in multiple tissues, such as muscle and the Y-organ, presenting a ubiquitous pattern. However, its expression peaked during stage II ovary development and became almost undetectable during the late stages (IV and V). This strict stage-specific expression pattern suggested that it likely played a role in initiating early ovary development rather than in ovary maintenance, which was comparable to the role of *amh* in initiating testis development in the yellow catfish (*Pelteobagrus fulvidraco*) [25,26].

The eyestalk ablation experiment provided indirect evidence for the function of *Eccyp307a1*. The eyestalk serves as a crucial neuroendocrine center in crustaceans, which can secrete various hormones, including gonad-inhibiting hormone (GIH) [27]. Upon unilateral eyestalk ablation in *E. carinicauda*, the inhibitory effect of GIH on the gonad was lifted, leading to a significant upregulation of *Eccyp307a1* expression. This indicated that the expression of *Eccyp307a1* could be regulated by inhibitory factors originating in the eyestalk. Its upregulation was one of the early molecular events signifying ovary activation and entry into a rapid developmental program. This finding was in agreement with results observed in other crustaceans after eyestalk ablation [28]. After the knockdown of *Eccyp307a1*, the gonadosomatic index and the number of follicle cells were both reduced. This experiment provided direct evidence for the role of *Eccyp307a1*. The observed phenotype of the RNAi group correlated with its expression during early ovary development. Based on these results, we could infer that if *Eccyp307a1* fails to express normally, subsequent key processes such as vitellogenin synthesis and deposition might be impeded, potentially causing ovarian developmental arrest.

### 3.2. The Function of Eccyp307a1 Exhibits Both Evolutionary Conservation and Species Specificity

In evolution, gene functions often produce variation due to the gene structure changes [29,30]. Our phylogenetic analysis showed that *Eccyp307a1* possessed a typical P450 domain and clusters with homologous genes from closely related species such as *M. nipponense* and *M. rosenbergii*, which reflected its evolutionary conservation within crustaceans. However, the *cyp307A1* proteins from crustacean and insect were on distinct clades, which suggested there was functional divergence among the different species. In insects, CYP307a1 was a key enzyme in synthesizing the molting hormone 20-hydroxyecdysone, which plays roles in governing growth, development, and metamorphosis [4]. However, other studies have also suggested that *cyp307a1* might play a role in reproduction [28,31]. Moreover, we noted that the function of *cyp302a1*, which belongs to the same subfamily, was also associated with reproductive development [32]. Our study indicated that *Eccyp307a1* functioned during early ovarian development in *E. carinicauda*. When we compared this to its molting-related role in insects [5], it is clear that the function of *Eccyp307a1* in *E. carinicauda* exhibited species specificity.

In summary, our study demonstrated that *Eccyp307a1* was regulated by the eyestalk and played an initiating role in early ovary development in *E. carinicauda* based on various experiments. Its function retained the core characteristics of P450 enzymes evolutionarily, while its specific biological role displayed adaptations unique to crustaceans. This discovery provided new clues for unraveling the molecular and endocrine regulatory network underlying ovary development in crustaceans.

## 4. Materials and Methods

### 4.1. Ethnic and Animals

This study was approved by the Ethics Committee of the Yellow Sea Fisheries Research Institute. All experiments were conducted in accordance with the Guidelines for the Care and Use of Laboratory Animals in China [33]. The animal study protocol was approved by the Institutional Animal Careand Use Committee of the Yellow Sea Fisheries Institute, Chinese Academy of Fishery Sciences (Approval Code: YSFRI-2024083; Approval Date: 20 August 2024). *E. carinicauda* were obtained from RiZhao Haichen Aquaculture Co., Ltd., Rizhao City, China. The animals were fed in our laboratory for 2 weeks and cultured in the presence of abundant oxygen at 25 °C and 30‰ salinity.

### 4.2. The Conservation Analysis

The amino acid sequence of *Eccyp307a1* and its homologous sequences in other species, including *Cherax quadricarinatus*, *Drosophila melanogaster*, *Eriocheir sinensis*, *Halocaridina rubra*, *Homarus americanus*, *Macrobrachium nipponense*, *Macrobrachium rosenbergii*, *Neocaridina denticulata*, *Penaeus chinensis*, *Penaeus monodon*, *Penaeus vannamei*, *Petrolisthes cinctipes*, *Portunus trituberculatus*, *Procambarus clarkii*, and *Sagmariasus verreauxi*, were downloaded from the public NCBI database. These sequences were then precisely aligned using multiple sequence alignment tools (http://www.ebi.ac.uk/Tools/msa/clustalo/ (accessed on 16 May 2025)), and the alignment results were exported.

### 4.3. Phylogenetic Tree Construction

The protein sequences of CYP307A1 from 16 species were downloaded from the NCBI database. All the protein sequences were imported into the TBtools-II software. A maximum likelihood method was employed for tree construction, which could generate a phylogenetic tree file. The tree file was subsequently imported into the iTOL (https://itol.embl.de/ (accessed on 8 August 2025)) tool for visualization and aesthetic refinement.

### 4.4. Tertiary Structure and Domain Analysis

The amino acid sequence of *Eccyp307a1* was submitted to the online tool Phyre 2.2 (http://www.sbg.bio.ic.ac.uk/phyre2/html/page.cgi?id=index (accessed on 10 June 2025)). Relevant parameters were filled according to the website’s requirements, and a Phyre search was performed to obtain the tertiary structure model of the *Eccyp307a1* protein. Specific domain analysis was conducted using Protein BLAST 2.17.0 of the NCBI database.

### 4.5. The Extraction of Total RNA

The tissues of adult shrimp and the larvae were flash frozen in liquid nitrogen. Then the samples were homogenized at a low temperature. For total RNA extraction, TRIzol reagent (Invitrogen, Waltham, MA, USA) was employed according to the manufacturer’s instructions. The RNA quality was determined via 1% agarose gel electrophoresis, and concentration was measured using a NanoDrop 2000 spectrophotometer (Thermo Fisher Scientific, Waltham, MA, USA).

### 4.6. RT-PCR and Real-Time PCR

The total RNA was reverse transcribed into cDNA using the ReverTra Ace reverse transcriptase kit (Toyobo, Osaka, Japan) according to the manufacturer’s protocol. Step 1: Reaction mixture: In total, 1 µg RNA, 4 µL 4× DN Master Mix, and DEPC-treated water were mixed to a final volume of 16 µL, and then incubated at 65 °C for 5 min. Step 2: Reaction mixture: The entire 16 µL product from Step 1 was mixed with 4 µL of 5× RT Master Mix II. The reaction conditions were as follows: 37 °C for 15 min; 50 °C for 5 min; 98 °C for 5 min; hold at 12 °C.

The resulting cDNA was diluted 5-fold and used for RT-PCR amplification. The reaction mixture contained 10 µL of enzyme mix, 2 µL of diluted cDNA, 0.4 µL each of forward and reverse primers, and 7.2 µL of H_2_O. Amplification conditions: 18S rRNA was used as the reference gene.

The qPCR experiment was performed using the SYBR Green Mix (Toyobo, Osaka, Japan). The reaction mixture comprised 10 µL of 2× SYBR Green Real-Time Master Mix, 2 µL of cDNA, 0.4 µL each of forward and reverse primers, and 7.2 µL of H_2_O. The reaction conditions were as follows: 95 °C for 3 min; 45 cycles × (95 °C for 15 s; 55 °C for 20 s; and 72 °C for 30 s); 65 °C for 0.06 s; and 95 °C for 0.5 s. Primers were designed using Primer5, and their sequences are listed in Table S1.

### 4.7. Eyestalk Ablation Experiment

Eighty healthy and vigorous female *E. carinicauda* shrimps were randomly selected and acclimatized for 7 days with sufficient oxygen, at 25 °C, and 30‰ salinity. They were then divided into two groups: a control group and an experimental group. For the experimental group, the right eyestalk was ablated, and the wound was disinfected with povidone-iodine solution. No treatment was administered to the control group. Throughout the experiment, shrimps were fed *Nereididae* normally. Samples were collected after 7 days. The expression of *Eccyp307a1* was measured via qPCR. The ovary was picked up for paraffin sections.

### 4.8. Paraffin Section and Histological Staining

Ovary samples were fixed in Bouin’s solution overnight, followed by washing in 70% ethanol. Then, the samples were dehydrated with a graded ethanol series, cleared in xylene, and infiltrated with paraffin wax. After embedding, the samples were sectioned at 7 µm thickness using a microtome. The sections were stained with hematoxylin and eosin (H&E). Briefly, sections were deparaffinized in xylene, rehydrated through a graded ethanol series, stained with hematoxylin for nuclei, and counterstained with eosin for cytoplasm. Finally, sections were dehydrated, cleared, mounted with neutral balsam, and observed under a microscope (OLYMPUS, Tokyo, Japan).

### 4.9. RNA Interference (RNAi)

The target sites for RNAi in *Eccyp307a1* were designed using an online tool siDirect version 2.1 (https://sidirect2.rnai.jp/design.cgi (accessed on 18 October 2024)), and the primers were listed in Table S2. Sixty healthy shrimps with undeveloped ovaries were raised at 25 °C and 30‰ salinity sea water with sufficient oxygen. They were divided into three groups: the experimental group, the negative control group, and the blank control group. The experimental group was injected with *Eccyp307a1* gene-specific siRNA at a dose of 1 µg/g body weight, while the control group was injected with NC siRNA at the same dose. A second injection of siRNA was administered on the third day post-initial interference. No treatment was administered to the control group. Six days after the initial interference, the body weight and gonad weight of each shrimp were measured. Samples were taken to detect the expression of *Eccyp307a1* and for histological sectioning.

### 4.10. Statistical Analysis

The statistical data of this study were plotted using Graphpad Prism 9 software and analyzed using Student’s *t*-test. * *p* < 0.05, ** *p* < 0.01, *** *p* < 0.001, **** *p* < 0.0001. ns: no significance.

## Data Availability

The original contributions presented in this study are included in the article/Supplementary Material. Further inquiries can be directed to the corresponding author.

## Supplementary Materials

The following supporting information can be downloaded at: https://www.mdpi.com/article/10.3390/ijms27031481/s1.
