# Supplementary material for: Functional Characterization of Eccyp307a1 in Early Ovary Development of Exopalaemon carinicauda
Source: Int J Mol Sci. 2026 Feb 2;27(3):1481. doi: 10.3390/ijms27031481 (PMC12898358; doi:10.3390/ijms27031481)
Supplement: Supplementary file 1 [file ijms-27-01481-s001.zip › ijms-4107527-supplementary.pdf]

Table S1 Primer sequence for RT-PCR and *real-time* PCR

Primer name	Primer Sequence (5'-3')	Primer Usage
Eccyp307a1-F	CAAGAACGAACCCGAGGAAT	RT-PCR, *real-time* PCR
Eccyp307a1-R	GAGAAATGGGACTGGGACAC	
18S-F	TGTATCTCAGGGCCTTGTCI	
18S-R	GACGTTTCCTGTACCTGGAC	

Table S2 Primer sequence for RNAi

Primer Name	Primer Sequence (5'-3')	Primer Usage
Eccyp307a1-A1	GATCACTAATACGACTCACTATAGGGCTATTGTTGTTATCCTTAATCTT	RNAi
Eccyp307a1-A2	AAGATTAAGGATAACAACAATAGCCCTATAGTGAGTCGTATTAGTGATC	
Eccyp307a1-B1	GATCACTAATACGACTCACTATAGGGTTAAGGATAACAACAATAGTGTT	
Eccyp307a1-B2	AACACTATTGTTGTTATCCTTAACCCTATAGTGAGTCGTATTAGTGATC	
NC-A1	GATCACTAATACGACTCACTATAGGGACGTGACACGTTCGGAGAATT	
NC-A2	AATTCTCCGAACGTGTCACGTCCCTATAGTGAGTCGTATTAGTGATC	
NC-B1	GATCACTAATACGACTCACTATAGGGAAACGTGACACGTTCGGAGTT	
NC-B2	AACTCCGAACGTGTCACGTTTCCCTATAGTGAGTCGTATTAGTGATC	
